# A review of different frying oils and oleogels as alternative frying media for fat-uptake reduction in deep-fat fried foods

**DOI:** 10.1016/j.heliyon.2023.e21500

**Published:** 2023-10-31

**Authors:** Niaz Mahmud, Joinul Islam, William Oyom, Kelvin Adrah, Samuel Chetachukwu Adegoke, Reza Tahergorabi

**Affiliations:** a Food and Nutritional Sciences Program, North Carolina Agricultural & Technical State University, Greensboro, NC, 27411, USA; b Department of Food Science and Technology, University of Georgia, Athens, GA, 30602, USA; c Joint School of Nanoscience and Nanoengineering, 2907 East Gate City Blvd, Greensboro, NC, 27401, USA

**Keywords:** Cooking oils, Deep-frying, Oil uptake, Oleogel, Oleogelator

## Abstract

**Purpose:**

This review aims to examine the potential of oleogels as a frying medium to decrease oil absorption during deep-frying and enhance the nutritional and energy content of foods. By investigating the factors influencing oil incorporation during deep-frying and examining the application of oleogels in this process, we seek to provide insights into using oleogels as an alternative to traditional cooking oils.

**Scope:**

Deep-frying, a widely used cooking method, leads to the retention of large amounts of oil in fried food, which has been associated with health concerns. To address this issue, researchers have investigated various methods to minimize oil absorption during frying. One promising approach is the use of oleogels, which are thermo-reversible, three-dimensional gel networks formed by entrapment of bulk oil with a low concentration (<10% of weight) of solid lipid materials known as oleogelators. This review will focus on the following aspects: a) an overview of deep-fried foods, b) factors influencing oil uptake and underlying mechanisms for oil absorption during deep-frying, c) the characterization and application of different frying oils and their oleogels in deep-fried foods, d) components of the oleogel system for deep-frying, and e) the health impact, oxidative stability, and sensory acceptability of using oleogels in deep-frying.

**Key findings:**

The review highlights the potential of oleogels as a promising alternative frying medium to reduce fat absorption in deep-fried foods. Considering the factors influencing oil uptake during deep-frying, as well as exploring the properties and applications of different frying oils and their oleogels, can result in improved product qualities and heightened consumer acceptance. Moreover, oleogels offer the advantage of lower fat content in fried products, addressing health concerns associated with traditional deep-frying methods. The capacity to enhance the nutritional and energy profile of foods while preserving sensory qualities and oxidative stability positions oleogels as a promising choice for upcoming food processing applications.

## Graphical abstract

**Figure:**
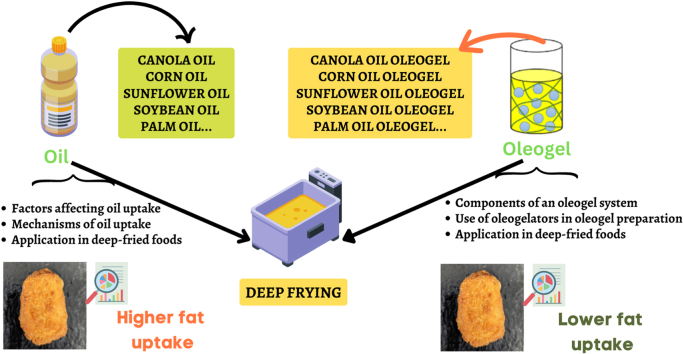

## 1 Introduction

Deep-fried foods have achieved widespread popularity globally, evident from the proliferation of fast-food outlets. Their sensory allure, boasting a distinctive fried flavor, golden-brown hue, and crispy texture, makes them highly enjoyable. However, a notable drawback is the substantial oil absorption during deep-frying. Our prior research on fried fish and chicken revealed significant increases in total fat content, with whiting fish fillets reaching 10.5% (from 1%) and chicken drumsticks escalating to 18% (from 2%) after deep-fat frying [1,2]. Similarly, the fat content of French fries can surge from 0.2% to 14%, while potato chips may reach as high as 40% fat content [3].

Numerous factors, encompassing heat and mass transfer mechanisms, alterations in product structure, temperature differentials between the core and crust, sample preparation, cooking duration, interactions between the frying medium and the product, and oil quality, collectively impact the absorption of oil [4]. The connection between excessive fat consumption and the escalating incidence of non-communicable diseases like obesity, heart disease, and diabetes mellitus, compounded by the complexities surrounding oil absorption, emphasizes the pressing need to investigate this phenomenon [5].

Numerous investigations have been carried out and are now being undertaken to discover novel approaches to decrease oil uptake during frying [6,7].

The type of frying oil and its composition can significantly impact oil absorption in deep-fat fried foods. Various oils possess distinct chemical properties, including differences in fatty acid composition and smoke point, which play a crucial role in their interaction with food during the frying process [8]. Although hydrogenated oils have been favored by many restaurants for their taste and crunch, their usage has decreased due to health concerns related to trans fats [9]. As a result, unsaturated oils have become the primary choice for deep-frying. However, using unsaturated oils may lead to greasy fried products with less crispiness and reduced shelf-life due to oxidation. Consequently, selecting the appropriate frying oil and understanding its behavior can help control and reduce oil absorption in fried foods.

Recently, Adrah et al. (2022) showed that fried chicken samples in oleogels had significantly lower fat-uptake than fried chicken in frying canola oil [6]. The oleogel technology transforms liquid oil into gel-like semi-solids. Oleogelators or structurants help turn liquid oil into a gel-like substance. The oleogel system develops networks that aid in trapping and holding onto oil molecules. Oleogels are a practical technique to structure polyunsaturated fatty acids (PUFA)-rich oils without compromising their health benefits [10,11]. However, the effectiveness of the oleogel system is influenced by the type of oleogelator employed as well as its network size, quantity, form, interactions, and temperature stability [12,13]. Consumer acceptance of oleogels hinges on their ability to imitate the properties of solid fats.

In conducting the review, we employed a systematic approach to gather relevant literature and ensure the rigor of the methodology. The primary databases used for literature search were PubMed, Scopus, and Web of Science, covering a range of articles published from 2010 to 2023. The keywords used for the search included “deep-frying,” “frying oils,” “oleogels,” “oil absorption,” “health impact,” “oxidative stability,” and “sensory acceptability.” We also conducted a manual search in key journals and reviewed the reference lists of relevant articles to find additional sources. The inclusion criteria were set to focus on research articles and reviews related to the use of oleogels for frying to reduce fat-uptake in deep-fried foods. Studies discussing the impact of different frying oils and their oleogels, health implications, oxidative stability, and sensory acceptability were prioritized for inclusion.

After identifying potential articles, we performed a thorough screening process to assess their relevance to our research objective. Any discrepancies in the selection were resolved through consensus with the research team. We then extracted pertinent data from the selected articles, including experimental methods, results, and conclusions, to synthesize the key findings. Therefore, this review aims to provide a comprehensive examination of the application of commonly used cooking oils in deep-frying, while also proposing the adoption of oleogel as a viable alternative to traditional cooking oils, with the intent of enhancing the overall quality of the end products.

### 1.1 Factors influencing oil uptake in deep-fried foods

#### 1.1.1 Size, shape, texture, and surface of the product

The amount of oil that a product absorbs while being fried is greatly influenced by its size and shape. Since oil absorption is mostly a surface event, product thickness has a negative correlation with oil uptake since most frying oil stays on the surface. According to a study, when the product thickness decreased, oil absorption significantly increased [14]. For example, French fries, having a smaller surface-to-volume ratio compared to potato chips, imply a linear relationship between product surface area and oil content. As a result, French fries absorb less oil than potato chips [[15], [16], [17], [18]]. The amount of oil that can enter the product is just about 1 mm [19]. Because most frying oil enters the product through the capillary pores in the crust, the structural characteristics of the food also influenced how much oil was absorbed. The cells that are disrupted during cutting are the best places for oil to absorb. Texture is also an important intrinsic factor influencing oil uptake in fried foods. The texture of a food product, particularly its surface roughness and porosity, plays a significant role in determining how much oil is absorbed during the frying process. Foods with a rough and porous texture tend to have more surface area exposed to the hot oil, which increases the likelihood of oil penetration and absorption. This can lead to higher oil uptake and result in a greasier and less desirable product. On the other hand, foods with a smooth and compact texture may have less surface area available for oil absorption, leading to lower oil uptake and a crispier texture. The amount of oil absorbed rises as a result of the cut surface’s increased roughness and contact with oil [20,21]. Therefore, using high-quality blades for cutting can lessen surface roughness, which in turn reduces oil absorption.

#### 1.1.2 Composition of the product

The original composition of the product is another crucial factor that influences the extent of oil absorption in fried products. The initial moisture and solid content of the product have the most significant impact on oil uptake during frying. Products with intermediate water content, including French fries and plantain cylinders, as well as those of high initial moisture content absorb more oil when they are fried. This might be a result of the relationship between oil intake and water loss [22]. It was shown that increasing the masa flour’s initial moisture level greatly boosted the final oil content of tortilla chips.

Additionally, the author concluded that the primary factor for oil absorption after cooling was the pore size distribution that occurred during frying. Large-scale moisture loss or a high starting moisture content were the major causes of the fried product’s very porous structure. When making tortilla chips, the amount of oil absorbed was correlated with the distribution of particle sizes in the dried masa flour. More oil-free tortilla chips were created with coarse masa flour than with fine masa flour. Oil absorption is limited by the coarse particles' ability to let water escape via the cracks they create [23,24].

Low-fat fried products are typically manufactured from ingredients with a high dry matter content. This characteristic becomes evident when we compare, for instance, French fries made from potatoes with a high dry matter content (>24%) to those crafted from potatoes with a lower dry matter content. The latter exhibited a 9% lower oil content, approximately 19.5%. When tubers possess a high dry matter concentration, the starch within them tends to develop a granular texture during frying, in contrast to its behavior when boiled [25,26]. It’s important to note that the incorporation of any leavening agents into the product also influences oil absorption. These outcomes were seen with battered and fried squid. The production of gas in the product, which the oil may be able to contain during frying, may be the cause of the enhanced oil absorption [27]**.**

#### 1.1.3 Oil type

An array of fats and oils, ranging from vegetable oils to animal fats, hydrogenated fats, and various combinations, are suitable for deep-fat frying. The selected frying oil should exhibit desirable frying characteristics, such as good fluidity, a neutral flavor, minimal frothing or smoke generation tendencies, low gum formation, and excellent oxidative stability within the fried food during storage. It should also be affordably priced [14].

When it comes to frying, common choices for oils include vegetable oils, sunflower oil, peanut oil, soybean oil, safflower oil, canola oil, and maize oil. Canola and peanut oils stand out with their high smoke points, which make them ideal for deep frying applications. Peanut oil is particularly favored due to its high content of polyunsaturated and monounsaturated fats, coupled with low levels of saturated fat. This composition makes peanut oil a healthier choice for frying compared to shortening and lard [28]. It’s worth noting that not all peanut oil is considered allergenic. Cold-pressed, expeller-pressed, and extruded peanut oils are recognized as allergens; however, highly refined peanut oil is generally safe for individuals with peanut allergies. In fact, highly refined oils like peanut and soybean are not classified as allergenic foods according to the Food Allergen Labeling and Consumer Protection Act [29].

An investigation was done to see how eight different frying oils affected how much fat fries absorbed [30]. Unsaturated fatty acids were shown to absorb more oil, but saturated fatty acids are more stable in frying applications [31]. Additionally, it was shown that the amount of unsaturation in frying oils enhanced the rate of oxidation. A superior frying medium, for instance, was discovered to be maize oil with less unsaturated fatty acids than soybean or canola oils with more unsaturated fatty acids. Additionally, it has been claimed that palm oil absorbs more oil than unsaturated cotton seed oil. The inconsistencies may be caused by the oil’s viscosity, which may also be connected to how well fats are absorbed [32].

#### 1.1.4 Degree of oil refinement

Crude oil undergoes a refining process to eliminate undesirable minor components that impart an undesirable taste to consumers while minimizing adverse impacts on the oil’s neutrality and refining yield. The objective is to eliminate any glyceride and non-glyceride molecules that could compromise the stability, color, flavor, and safety of the refined oils. These problematic substances primarily include phosphoacylglycerols, volatiles, pigments, free fatty acids, and pollutants. It is important to note, however, that not all minor components present in fats and oils are harmful. For example, phytosterols are nutritionally valuable, and tocopherols, which contain vitamin E action, shield the oil from oxidation. Therefore, all phases of the refining process should be completed with the least amount of loss of desired compounds in order to get the highest oil quality. The smoke point of an oil increases with refinement. This is so that the contaminants that may make the oil smoke can be eliminated during refining. The oil’s greater smoke point corresponds to its lighter hue, according to a straightforward rule of thumb [14].

#### 1.1.5 Quality of frying oil

The frying medium is chosen based on its cost, effectiveness, taste, sensitivity to oxidation, and availability. Deep-fat fried products absorb different types of byproducts and residues depending on the oil’s composition. Although there is a positive correlation between frying duration and oil absorption and deterioration, this connection is not linear. It was discovered that the oil taken out of the fried product contains more polymers than the oil still in the fryer. It was noted that when the rate of oil degradation increased, the interfacial tension between the product and frying oil dropped.

#### 1.1.6 Frying temperature

In order to guarantee the high quality of fried foods, one of the most crucial parameters that must be regulated during frying is the frying temperature. A difference in frying temperature is linked to oil deterioration, increased surfactant production, product discoloration, and oil absorption. Regarding the impact of frying temperature on oil uptake, researchers have found conflicting findings. According to one study, moisture loss and oil uptake were unaffected by frying temperature [33].

On the other hand, another study found that oil absorption was higher at lower frying temperatures [34]. This may be because at lower frying temperatures, the food tends to stay in the pan for a longer period of time, increasing oil absorption. The dehydration process was also induced by increased frying temperatures, and the initial crust development served as a barrier to oil absorption, preventing moisture from the food product from escaping and consequently impeding oil uptake. Additionally, it was shown that there was no discernible oil absorption at frying temperatures between 150 °C and 180 °C but that rising frying temperature causes a decline in oil uptake [14].

#### 1.1.7 Frying time

Both the frying time and temperature have an impact on how much heat is delivered to the product, therefore they are interrelated. The product usually tends to have increased oil absorption when the frying duration is longer than the ideal at a constant temperature. When frying potatoes, it was discovered that the oil content was twice the square root of the frying time [16,33,35]. It was also found that increased frying time increases the crust’s porosity, which leads to greater oil absorption while frying instant fried noodles [36].

### 1.2 Mechanism of oil absorption in deep-fried foods

Three theories including water displacement, cooling-phase effect, and surface-active agents have been proposed to explain the complicated process of oil absorption [4,21].

#### 1.2.1 Water replacement

During deep-frying, this process proposes replacing water with oil. When a food product is exposed to a high temperature frying medium, fast evaporation occurs within the surface, resulting in the creation of a crust. The moisture in the product’s core is turned to steam, resulting in a positive gradient. Gaps, open capillaries, flaws, and fissures in the membranes and cellular structure allow steam to leave the product’s core. Fat attaches to the product’s surfaces as the process progresses, eventually migrating to fissures and spaces caused by moisture evaporation and frying. Due to the lack of resistance and the relatively high size of the voids, fat flows readily inwards [37,38]. This process might explain the link discovered in multiple investigations between oil uptake and water loss in deep-fat fried products. The oil enters the product’s pores and helps to retain structural integrity by avoiding the collapse of pores formed by evaporation. However, with some formulations, the oil may not be able to fill the holes and pores generated until the water has entirely evaporated. This is due to the resistance formed by the inner steam pressure during frying, which prevents oil absorption into the product’s core. This phenomenon has been thoroughly researched, and it has been discovered that fat absorption occurs mostly when the product is cooling [39].

#### 1.2.2 Cooling phase effects

The absorption of oil after it has been removed from the frying medium is described by this suggested process. Water vapor condenses as it is released from the frying medium, lowering the vapor pressure of the pores in the crust [37]. The reduction in pressure induces the flow of oil into these pores. Consequently, achieving a balance between adhesion forces and oil drainage during the cooling phase becomes essential for oil absorption using this method [21]. Therefore, it is possible to examine the microstructure of the crust developed during deep-fat frying to evaluate oil absorption and distribution. According to Ziaiifar et al. (2008), the viscosity of oil rises as frying time increases [21]. The increasing viscosity is due to the oil polymerization. In effect, the surface characteristics of the product and the viscosity of the frying medium are the two key parameters that determine oil absorption. To control oil absorption during the cooling phase, superheated hot air or steam is used to blast away the surface oil, or an adsorbent paper is used to soak the oil from the fried product’s surface [39,40].

#### 1.2.3 Surface-active agents

Deep-fat frying causes a sequence of chemical processes that cause oil quality to deteriorate [41]. Typical reactions that occur during the frying result in the formation of a variety of chemicals. The link between glycerol and fatty acids is cleaved by hydrolytic processes initiated by the adulteration of the oil phase with water. The formation of free fatty acids, monoglycerides, and diglycerides is accelerated by increasing the frying temperature [42]. These polar chemicals and surface-active substances (mono- and diglycerides) boost the frying medium’s foaming propensity, which speeds up the hydrolytic process. The creation of these degradative chemicals influences the oil-food contact, lowering surface tension between the two components. Excessive oil absorption occurs when the surface tension is low, and the contact duration is extended. The production of surfactants has an impact on heat transmission at the oil-food contact. Continuous heat transfer to the food induces drying at the food surface and increases water migration to the food’s exterior [38].

## 2 Characterization and application of most commonly used frying oils in deep-fried foods

Frying positively impacts food properties by improving its juiciness, crispiness, and color properties. Although frying involves immersion cooking in oil at elevated temperature, a very essential step to undergo to ascertain the physicochemical and nutritional attributes of the final product would be to determine the characteristics and quality of the frying oil. Due to the paucity of the technological applications of vegetable oil-which can be ascribed in part to their specific physicochemical properties, modifications to increase their use as an edible frying medium is gaining attention.

**Fig. 1:**
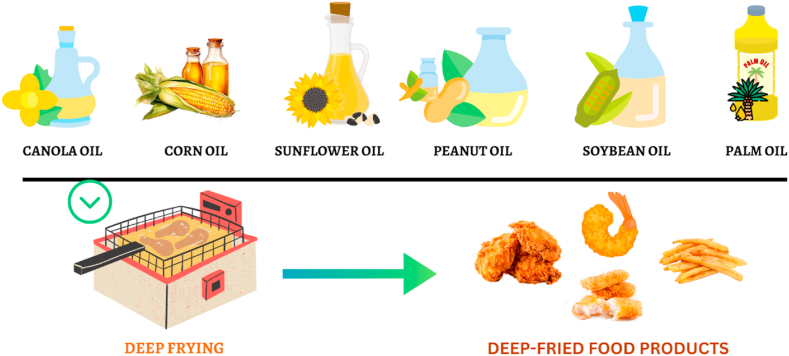
Depiction of frequently used frying oils and their use in deep-frying operations.

Although techniques such as interesterification, hydrogenation, blending, and fractionation have been used to enhance oxidative stability and textural properties [43] of vegetable oil, the United States Department of Agriculture has recommended the consumption and use of non-hydrogenated, low saturated, and high poly and mono-unsaturated fats such as olive, palm, corn, canola, sunflower oils, etc. [44] in food preparations. Vegetable oils rich in unsaturated fatty acids are prone to oxidation when exposed to catalytic systems like metals, heat, light, enzymes, and microorganisms. This process can result in the formation of intricate reactions, including photooxidation, autoxidation, and enzymatic oxidation [45]. Characteristics of different frying oils are presented in Table 1. Additionally, the various types of frying oils used in deep frying are presented in Fig. 1.

**Table 1:** Characteristics of different frying oils.

Properties	Canola oil [46]	Corn oil [46]	Safflower oil [47]	Sunflower oil [47]	Peanut oil [48]	Soybean oil [49]	Palm oil [50]	Rice bran oil [51]
Source	Canola seeds	Corn seeds	Safflower seeds	Sunflower seeds	Peanuts	Soybeans	Palm fruit	Rice bran
Composition	High MUFA, low SFA	High PUFA, low SFA	High PUFA, low SFA	High PUFA, low SFA	High MUFA, low SFA	High PUFA, low SFA	Low MUFA, high SFA	High MUFA, low SFA
Solidification temperature	Liquid at room temp	Liquid at room temp	Liquid at room temp	Liquid at room temp	Liquid at room temp	Liquid at room temp	Solid at room temp	Liquid at room temp
Melting point	−8 to −5 °c	−15 to −10 °c	−15 to −10 °c	−17 to −14 °c	−4 to -1 °c	−10 to 7 °c	35 to 40 °c	24 - 27 °c
Stability	Moderate stability	Moderate stability	Moderate stability	Moderate stability	High stability	Moderate stability	High stability	High stability
Reusability	Yes	Yes	Yes	Yes	Yes	Yes	Yes	Yes
Smoke point	High	High	High	High	High	High	Medium-high	High
Foam formation	Low	Low	Low	Low	Low	Low	Low	Low
Shelf life	Moderate	Moderate	Moderate	Moderate	Moderate	Moderate	Long	Long
Oil absorption	Moderate	Moderate	Moderate	Moderate	Moderate	Moderate	High	Moderate
Nutritional content	Omega-3 & omega-6	Omega-3 & omega-6	Omega-6	Omega-6	Vitamin E	Omega-3 & omega-6	Vitamin E	Vitamin E
Techno-functional performance	Standard deep-frying characteristics	Standard deep-frying characteristics	Standard deep-frying characteristics	Standard deep-frying characteristics	Standard deep-frying characteristics	Standard deep-frying characteristics	Suitable for high-temperature applications	Standard deep-frying characteristic

### 2.1 Canola oil

Canola oil is characterized by high concentrations of monounsaturated (MUFA) and polyunsaturated fatty acids (PUFA), including a balanced ratio of omega 3 to 6 PUFA, and small amounts of saturated fatty acids (SFA) compared to other oils such as soybean, and sunflower oil [52], also plant sterol and tocopherols are present in detectable concentrations-all which have been reported to confer cardioprotective benefits [53]. However, its uses are confined to confectionary coating, frying operations, center fillings, whipped toppings, and coffee whiteners [52].

Limitations in specific physicochemical properties such as oxidative stability have necessitated different modification approaches which will yield oil varieties with tailored applicability. The presence of linolenic acid, chlorophyll, and its degradation products including minute amounts of fatty acids possessing more than three double bonds with known high chemical reactivity can induce oxidation in canola oil. Bearing this in mind, since frying operations require elevated temperatures, canola oil containing 85% oleic acid and 2% linolenic acid was formulated specifically for frying with superior oxidative stability [52]. Oil uptake of French fries, chicken thigh, cheese samosa, breaded chicken, and French toast deep fried in canola, soybean, corn, and sunflower oil was investigated [54]. The least amount of oil uptake was reported in cheese samosa (10%) and French fries (12%) using canola oil, while soybean and corn oil displayed high oil uptake in French fries 23 and 24%, respectively, and even higher amounts in cheese samosa 27 and 28% [54]. The authors attributed the higher amount of oil uptake recorded for soybean and corn oil could be related to the density, viscosity, fatty acid composition, and surface tension of the respective oils coupled with the microstructural integrity of the product. For instance, the oil drainage rate after frying and mass transfer rate during the cooling phase is characteristics of oil uptake that can be directly influenced by oil density [55].

Although the densities of conventional oils are well established within temperature range of 110–140 °C but frying typically occurs at 150–180 °C [56]. Therefore, oils with higher density will exhibit slower migration especially after frying leading to increased oil uptake. The estimated molar volume of vegetable oil can be predicted by the modified Racket equation which is dependent on the molecular weight and critical properties of individual fat present in the pure oil sample. It is worth mentioning that mathematical models accurately describing the transport rate at frying temperatures are still unavailable. However, the proportion of PUFAs such as eicosenoic acid (C20:1) and octadecanoic acid could change the relative density of canola oil [52] essentially impacting the amount of fat taken up by the food when canola is used as a frying medium [57].

Canola oil’s viscosity at 20 °C is reported to be 78.2 mPa s [52] and at a 100% increase in temperature i.e. 40 °C it dropped to 34.9 mPa s [55] while at a frying temperature of 180 °C it was drastically reduced to 3.0 mPa s indicating a finite influence of viscosity on oil uptake mechanism. French toast deep-fried in canola oil was shown to have the lowest oil uptake compared to soybean, corn, and sunflower oils [54]. This may be attributed to their respective viscosities. For instance, soybean (3.2 mPa m), and corn oil (3.5 mPa m) both have higher viscosities than canola oil (3.0 mPa m) at 180 °C. This was corroborated by the report of [58] where the fat uptake of canola oil deep-fried French fries was the lowest. Similarly, pre-dried fries without coating (control) deep-fried for 90 min exhibited a similar reduction in fat content (19%) compared to fries coated with 2% methylcellulose and guar gum [59].

The smoke point is the temperature at which the smoking process initiates, serving as an important indicator of frying stability. Beyond this smoke point, oil starts to decompose into its individual free fatty acids and glycerol. Glycerol, in turn, undergoes further breakdown into acrolein, a significant component of the smoke. The irritation caused by this smoking process signifies the beginning of nutritional deterioration and flavor degradation. It’s worth noting that the smoke point can vary considerably based on the oil’s source and level of refinement. For instance, canola oil has been documented to have a smoke point of 204 °C [60].

### 2.2 Corn oil

Corn oil is an all-purpose cooking oil possessing unique flavor attributes and superior oxidative stability compared to other vegetable oils, it contains about 15% SFAs, 25% MUFAs, and 60% PUFAs respectively [61]. Factors such as growing season, genotypic expression, and climatic conditions are used to predict the fatty acid composition of corn oil.

Generally, corn oil is categorized among oils with high amounts of oleic C18:1 ω-9 (20–42.2%), linoleic C18:2 ω-6 (34–65.6%), and α-linolenic acid C18:3 ω-3 (0.5–1.5%) [61,62], however, they have found excellent and consistent use in high-temperature application such as deep fat frying. Corn oil contains about 1.3%–2.3% of unsaponifiable material consisting mainly of squalene, tocopherol, tocotrienols, and phytosterol. Corn oil boasts exceptional oxidative stability due to its abundant presence of natural antioxidants, including ferulic acid, ubiquinones, tocotrienols, and tocopherols. These inherent stabilizing qualities elevate corn oil to a position of great significance in industrial frying processes [61].

A study by Ref. [63] reported that the fat content of corn oil deep-fried chicken samples was lower than the canola oil deep-fried chicken. Additionally, higher puncture force and lower thiobarbituric acid reactive substances (TBARS) levels were seen when corn oil was used for frying. Another study observed that corn oil used to fry chicken thighs absorbed the least oil compared to other vegetable oils [54].

The speed of the oil movement across the food surface is dependent on the fatty acid composition, density, viscosity, and surface tension of the frying oil. The denser, and more viscous the oil is at frying temperature, the more uptake occurs irrespective of the molecular nature of the food matrix. At room temperature, the average density, viscosity, and surface tension of corn oil are reported to be in the range of 915.3–925 kg/m^3^, 59.2 mPa m, and 31.6–32.2 mN/m [55,62]. However, at frying temperature (180 °C) these values are exponentially reduced suggesting decreased oil migration.

Although the smoke point of corn oil is 230 °C, which is comparable to other commodity vegetable oils, by-products of elevated frying temperature such as monomeric, dimeric, polymeric acylglycerols and fatty acids are more specific indicators of oil degradation compared to smoke point. Smoke point is therefore an important criterion for the selection of fats for deep frying operation (deep-frying requires fat and oil with high smoke points) [62].

### 2.3 Safflower and Sunflower oils

Safflower (*Carthamus tinctorius* L.) oil is popularly used in many parts of the world as edible cooking oil due to its high content of MUFAs and PUFAs [64]. Among commercial vegetable oils, safflower oil exhibits the highest level of linoleic acid 71–79.5%, while SFAs, such as stearic and palmitic acids are present in relatively low concentrations [65]. Palmitic, stearic, and oleic acids have been reported to be present in safflower oil in the following range 6–8.6%, 2–3%, and 16–20%, respectively [64,66].

Sunflower oil, similar to safflower oil contains a relatively high amount of linoleic acid (70%) which makes it highly susceptible to oxidation, especially during deep-frying operations [67]. Deep fat frying can modulate the fatty acid composition of frying oils essentially resulting in inflections in fat uptake. This was verified by the quantification of fatty acid composition before and after deep frying potato chips in refined linoleic sunflower oil (RLSO) and refined oleic sunflower oil (ROSO) [68]. In this study, linoleic acid in RLSO and ROSO was 54.30% and 15.40% before deep fat frying, while after deep frying, the linoleic acid content reduced to 46.50% for RLSO and 11.05% for ROSO with a concomitant increase in SFAs such as stearic and palmitic acids [68]. This lends credence to the idea that such modulation can result in slower oil drainage and high viscosity leading to increased oil uptake. In a simulated study, high oleic sunflower oil exhibited an absorption peak at 2.9 μm after deep frying at 185 °C, the absorption peak was absent prior to frying [67]. The authors posited that the absorption peak was due to the onset of oxidation caused by the formation of intermolecular hydrogen bonds of hydroxyl groups. In a similar study, sunflower oil was used to deep-fry potato strips at 150, 170, and 190 °C, respectively [69]. The oil uptake was reduced with an increase in frying temperature, which can be correlated with the changes in the fatty acid profile of the oil. For instance, at 150 °C, the authors reported a fat uptake of 0.310% with a relatively high concentration of heavy molecular weight fatty acids such as linoelaidic acid (C18:2) and methyl lignocerate (C24:0) while at 170 °C the fat uptake reduced to 0.236% in association with the above-mentioned fatty acids. Since mass transfer is characterized by the movement of water in the form of vapor from the food matrix into the oil, subsequently followed by the migration of oil into the void vacated by the water vapor [55]. It is, therefore, logical to assert that the migration of heavy molecular weight fatty acids affects both mass transfer during deep frying and oil drainage rate after frying i.e., the fatty acid composition is directly linked to oil viscosity and density – the two most important parameters governing oil absorption and drainage.

The proportion between eicosenoic (C20:1) and octadecanoic acid plays a crucial role in relative density fluctuation observed in vegetable oil [52] and will by extension affect the fat uptake in deep-fried foods. The viscosity and density of sunflower oil at 40 °C is reported to be 35 mPas and 900 kg/m^3^ [70]. Compared to sunflower oil, corn (34.5, 904.4 kg/m^3^), and soybean (31.3, 903.3 kg/m^3^) oils have higher densities at a similar temperature, while canola (34.9, 901.7 kg/m^3^), and peanut (38.8, 899.3 kg/m^3^) exhibited similar densities to sunflower oil. Since viscosity is dependent on factors like oil quality, frying temperature, and oil type [55], sunflower oil may exhibit lower fat uptake compared to vegetable oils such as peanut and olive oil with higher viscosities at 40 °C.

On the contrary, at frying temperature (160 °C), the viscosity of sunflower oil was reported to be within the range of 7–9 mPa s [71], while at the same temperature the viscosities of peanut, olive, corn, and soybean oils are 4.0, 4.0, 4.3, and 3.9 mPa s, respectively [55]. This implies that sunflower oil at frying temperature will exhibit slower oil drainage resulting in increased oil uptake. This is further contextualized when French toast and chicken thighs were deep fried in canola, corn, sunflower, and soybean oils, respectively, sunflower oil showed the highest fat uptake [54]. Similarly, potato strips deep-fried in sunflower oil showed approximately 12% oil uptake [72].

### 2.4 Peanut and soybean oils

Peanut (*Arachis Hypogaea* L.) oil is a commodity vegetable oil that is abundant in natural vitamin E with an almost equal amount of α-tocopherol and γ-tocopherol [73]. It is replete with a high concentration of MUFAs and PUFAs (about 80%) of which oleic (C18:1) and linoleic acid (C18:2) form the major part as well as SFAs such as palmitic and stearic acids (20%) [74].

Peanut oil contains about 96% triacylglycerol of which the major ones are (Palmitic acid, Oleic acid, Oleic acid)-POO (8%), (Oleic acid, Oleic acid, oleic acid)-OOO (10%), (Linoleic acid, linoleic acid, Oleic acid)-LLO (12%), (Palmitic acid, Linoleic acid, Oleic acid)-PLO (13%), and (Oleic acid, Oleic acid, and Linoleic acid) OOL (17%), while eicosenoic (C29:1), arachidic (20:0), lignocerate (C24:0), and behenic acid (22:0) are present in little amounts [74]. Peanut oil has found excellent use in cooking and frying operations due to its superior oxidative stability compared to other vegetable oils such as olive, soybean, and sunflower oil. The fat uptake of deep-fried potato strips in 10 different oils was investigated [58]. In this study, high oleic sunflower oil followed by peanut oil had the lowest oil uptake in finished fried potato strips. A similar result was reported by Ref. [58]. The presence of high molecular weight SFAs such as lignoceric acid (C24:0) albeit in small amounts, can impact the viscosity, and density of peanut oil during frying.

Heat transfer rate could leverage the molecular weight of individual fatty acids to increase the fat uptake of food samples. When food samples are deep-fried, the heat transfer coefficient strongly affects the surface temperature more than the core temperature. An appreciable increase in the heat transfer coefficient will result in elevated heat flux from the oil to the food causing a higher surface temperature [75]. This higher surface temperature, especially in thin samples such as potato strips, will result in a higher crust thickness. Oil migration into the core of the food sample is facilitated by the relative molecular weight of the fatty acid component of the oil and the crust thickness.

The fatty acid composition of soybean oil changes with seed oil deposition and maturity, lipid components such as triacylglycerols, linolenic acid, and palmitic acid content of soybean oil decrease with maturity, while the oleic acid content increases [76]. Untreated soybean oil is susceptible to oxidation, hence its rarity in both domestic and industrial deep-fat frying operations. However, soybean oil with less than 3% linolenic acid was reported to exhibit increased stability during deep-frying operations.

A study was conducted to test the effect of deep-fat frying of potato strips and chicken on the quality of soybean oil [77] reported a 6.9% fat uptake for potato strips while the deep-fried chicken surprisingly did not take up fat. Deep fat frying operations can significantly impact the fatty acid compositions of the frying oil. When soybean oil was used as a frying medium at 180 °C, the oleic and linoleic acid content of the oil reduced significantly, correspondingly saturated fatty acids such as (C16:0), C18:0, and (C20:0) significantly reduced after frying [78]. In addition to the theory of thermal degradation of the frying oil-which may have converted these fatty acids into volatile compounds such as furans, aldehydes, alcohols, ketones, alkanes, etc. [79], we speculate that the unaccounted portion of fatty acid may have been taken up by the food sample. The rate of migration of soybean oil is governed by their kinematic viscosity and density which increases with increases in polymerized triacylglycerols [80]. In a more recent study involving ten different vegetable oils, French fries deep-fried in soybean oil showed the highest fat uptake [58].

### 2.5 Palm oil

Palm oil is extracted from the ripened mesocarp of the fruit of the oil palm tree (Elaeis guineensis).Palm oil, with its unique fatty acid (FA) and triacylglycerol (TAG) composition, finds versatile applications in the food industry [81]. Notably, it stands as the sole vegetable oil containing nearly equal proportions of both saturated and unsaturated fatty acids. Crude palm oil (CPO) serves as a source of vitamins and plays a vital role in culinary and frying practices. Fractionation yields two primary products, palm olein, the liquid fraction of CPO, and palm stearin, the solid fraction, each endowed with distinct physicochemical attributes.

Numerous culinary and commercial products, including cosmetics, shortenings, candles, ice cream, lubricants, biodiesel, and toothpaste, contain CPO, palm olein, and palm stearin as significant ingredients. By supplying solid fat functionality without the need for hydrogenation, palm stearin helps lower the consumption of trans fats in diets [82,83]. Among frying oils, palm oil comes in front. Its distinct fatty acid makeup and high smoke point, which is around 230 °C, are further characteristics. A study examined how well palm oil and palm olein performed during frying. The acceptability and stability of palm oil in batch fryers for restaurants and continuous frying in the production of snack products were the main topics of the evaluation [84]. The article showed that when compared to high oleic oils, the frying performance of palm oil and its byproducts were comparable [84]. The quality grades and the benefit of using palm oil in deep frying depend on the quality of the extracted palm oil. In general, high-grade palm oil is defined as having low free fatty acids (FFA) and moisture content, very low levels of contaminants, and an excellent bleachability index. The utilization of palm oil is determined by its quality. A good-grade oil comprises more than 95% neutral TAGs and 0.5% or less FFA. High-quality oil is utilized in the edible oil sector, whereas lower-quality oils are used in the non-edible industry for biofuels, candles, cosmetics, and soap [85]. Refined oils must adhere to an industry standard that calls for a 0.1% FFA content. FFA is often found in crude oils around 1–3%. Physical refinement is advised when the oil contains a high FFA content [86,87]. Physical refining of CPO at a pressure of 0.8 kPa decreased FFA from 12% to 1.3% at 220 °C and 0.5% at 230 °C [88]. It is advised to use lower deodorization temperatures [89].CPO has a distinct fatty acid content (FAC) when compared to other vegetable oils.

The most commonly utilized analytical method in lipid research is Fatty Acid Composition (FAC) analysis. Crude Palm Oil (CPO) naturally exhibits a semi-solid state at room temperature due to its nearly equal proportion of saturated fatty acids (SFA) and unsaturated fatty acids (UFA). A significant component of the FAC in CPO is palmitic acid in the C16:0 form, present in high concentrations. Additionally, oleic acid (C18:1), linoleic acid (C18:2), and stearic acid (C18:0) are noteworthy, accounting for 40%, 10%, and 5%, respectively, of the total composition [90]. Similar to other vegetable oils, CPO consists of mixed triacylglycerols (TAGs) and partial acylglycerols, including diacylglycerols (DAGs) and monoacylglycerols (MAGs). The hydrolysis byproducts of TAGs, DAGs, and MAGs can influence the oil’s melting point and crystallization behavior [91]. When partial acylglycerol concentrations exceed 10%, particularly when temperatures fall below 20 °C, cloudiness might result [92]. According to research, the range of TGAs in CPO and fractions is 94–98%; DAGs are 8–8%; MAGs are 0.21–0.34%; and FFAs are 2–5%. Inadequate handling and storage conditions might cause a hydrolytic breakdown, which can result in higher readings [93]. Lard and beef tallow have mainly been replaced by palm oil in large-scale industrial frying. French fries, fried chicken, instant noodles, snack foods, and chicken fillets are among the foods that are commonly cooked with palm oil and palm olein [94,95]. Fast food establishments also utilize a significant amount of palm oil for frying [84]. According to research, palm olein’s oxidative stability during frying was equivalent to that of high oleic vegetable oils, hydrogenated sunflower and cottonseed oils, and other oils. Palm oil is not easily oxidized, polymerized, or foamed. In the fryer, palm oil does not leave behind any gooey or sticky residues [84].

According to research [96], palm oil is the oil of choice for the leading snack food makers in several European Union (EU) nations due to its excellent performance and great oxidative stability. The most widely used commercial frying oil is palm olein, which has a lower melting point than CPO (22–24 °C) and prevents the mouthfeel of fried products from becoming waxy or greasy [87]. According to another study, palm olein performed similarly in frying as high-oleic, low-linolenic canola oil [97]. According to reports, palm oil has a 12-day useable life when used as a frying medium. Filtration and refilling of the oil with new oil samples may be part of the management of the frying oil [98]. The form, kind, texture, and microstructure of the product all affect how the oil and nutrients diffuse in the fried result. Additionally, it is influenced by the oil’s viscosity, frying temperature, and cooking time. The premise behind the use of palm oil in frying is not only that it is stable during the frying process but also that the tocopherols, phenolics, tocotrienols, and carotenoids components will migrate into the fried products [99].

### 2.6 Rice bran oil

Several Southeast Asian nations generate a significant amount of rice. Additionally, the United States, Europe, and Latin America also generate substantial amounts. India is the next-largest producer of rice, followed by China. The extraction of rice bran, a byproduct of the rice processing industry, results in rice bran oil. Each kind of rice bran has a different oil content, and the techniques and circumstances attained during rice milling have an even bigger impact. As a result, there is 15–25% oil in rice bran. Around 6–7 million tons of food-grade oil might be produced from the approximately 4000 million tons of paddy produced globally. The oil and other byproducts produced by modern recovery methods would be of higher quality than those produced by the outdated technology now in use in China, India, and other Asian nations [100].

Due to the availability of natural bioactive phytoceuticals, such as -oryzanol, tocopherols, and tocotrienols (tocols), rice bran oil (RBO) is one of the most nourishing and healthy edible oils [101,102]. These compounds also play significant roles in the prevention of several illnesses. Due to its high smoke point, stability, and special frying qualities, RBO is more often used as frying oil since it uses less oil while frying than other oils [103]. Due to its high amount of tocopherols and tocotrienols (∼860 ppm), refined RBO plays a significant role as a superior salad and frying oil with good oxidative stability [104].

The heat stability of frying oil is primarily determined by two elements: the fatty acid content and the presence of antioxidants and antioxidant precursors. Frying oil should contain low levels of polyunsaturated fatty acids, such as linoleic or linolenic acids, and high levels of oleic acid. Additionally, moderate levels of saturated fatty acids should be present. For instance, rice bran oil consists of approximately 30% linoleic acid, 44% oleic acid, and 23% saturated fatty acids. Unsaturated fats are prone to thermal breakdown from oxidation during cooking or heating [105], which can result in a variety of chemical changes such as oxidation, polymerization, pyrolysis, and hydrolysis [106]. According to Ref. [107], rice bran oil has a large amount of unsaponifiable matter and gamma oryzanol. Tocopherols, tocotrienols, phytosterols, polyphenols, and squalene are examples of unsaponifiable. Natural compounds such as squalene, sterol fraction, and quercetin have reportedly been found. Vegetables are more stable at higher temperatures because of oryzanol and ferulic acid [108]. Rice bran oil’s low viscosity makes it easier to cook food without using as much oil [109]. Due to its high smoke point and delicate flavor, it is considered to be a good frying/cooking oil [110]. Oil’s frying stability may be evaluated by keeping an eye on the physicochemical changes that take place while it is heated to temperatures between 180 ± 2 °C.

## 3 Oleogel as an alternative to frying oil

Various techniques have been investigated to minimize oil absorption during deep-frying [111]. One of the recommended solutions to address the challenge of lowering oil absorption and improving the nutritional and energy content of fried foods is to modify the frying medium into oleogel [112]. Oleogel is a semisolid system, with a continuous phase prepared with a hydrophobic liquid (edible oil) where a self-assembled network (composed by structurant/oleogelators) entraps the hydrophilic liquid to form a thermo-reversible three-dimensional gel network [113]. Oleogel exhibits viscoelastic qualities like solid fat and has been utilized in many studies as a modified frying media to limit oil absorption in deep-fried foods. Additionally, oleogel can provide nutritional benefits while enhancing the sensory and technical properties of food products [114,115].

### 3.1 Components of an oleogel system

The formation of oleogels depends on two crucial components: the organic solvent and organic gelators. In the food sector, generally edible oil is used as the organic solvent for the development of oleogels. However, various structurants, including plant waxes, monoglycerides, ethylcellulose, phospholipids, and phytosterols, have been authorized for use as oleogelators for the preparation of food-grade oleogels [111]. However, the type of olegelators used for preparing oleogel greatly influences the properties of oleogel. Conversely, surface activity, thermoreversible characteristics, natural origin, and Generally Recognized as Safe (GRAS) status are significant criteria that render oleogels suitable for use in the food industry [116].

#### 3.1.1 Oil

In the process of developing oleogels, the composition of both the gelling mediator and the gelling solvent has a significant impact on the thermal, textural, and rheological properties of the oleogels. The role of the oil phase in the oleogel system is significant, as it has a significant impact on gel behavior and the uptake of lipid droplets or bioactive in the gastro-intestinal phase. The gelation reaction of different oleogel systems is determined by the chemical structure of the oil, namely the length of the fatty acid chain and the quantity of unsaturated fatty acids. Ref. [117] evaluated five different oils (rapeseed, rice bran, corn, sunflower, and high oleic sunflower oil) with variable levels of saturation and unsaturation to investigate the effect of fatty acid composition on the gelling ability of wax. The authors reported that oils with a higher saturation level strengthen the oleogel.

On the other hand, oil viscosity and polarity appear to have a significant impact on the gelation kinetics and crystallization behavior of oleogels. It has been found that the interaction between the gelator molecule and oil in ricinoleic acid-based oleogels was improved as oil polarity increased, resulting in a decrease in gel strength [118]. In contrast, ethylcellulose (EC) oleogels exhibit an increase in gel hardness with increasing polarity due to an increase in EC solubility during heating [119]. De Vries, Gomez, van der Linden, & Scholten, (2017) studied the effect of protein aggregates on oleogels made with three different oils (extra virgin olive oil, sunflower oil, and castor oil). The researchers noted that oils with higher polarity tend to produce weaker gels, primarily because of the strong interactions between the gelator and the solvent.

Long carbon chains and increased viscosity form gels with increased firmness. In addition, a higher degree of unsaturation in the oil phase leads to the production of a more crooked spatial arrangement, which increases the hydrophobicity of oil by decreasing interaction energy. This increased hydrophobicity promotes the solubilization of non-polar structurants, facilitates the creation of a large number of connection zones, and consequently produces gels with greater strength [120]. Zetzl et al. (2014) examined the effect of oil type (canola oil, soybean oil, and flaxseed oil) on the rheological and microstructural characteristics (pore diameter) of oleogels and reported that canola oil produce pore with a higher diameter (more stable gel), but pore diameter decreased as the unsaturation level of the oil component increased. Thus, it can be concluded from the preceding discussion that oils with lower polarity, a higher degree of unsaturation, and greater viscosity are more appropriate for creating firmer oleogels.

#### 3.1.2 Oleogelators

##### 3.1.2.1 Low molecular weight oleogelators (LMWOGs)

These oleogelators consist of low-molecular-weight chemicals that self-assemble in order to form a stable crystal network that stabilizes the resulting oleogel. The physical interactions responsible for network development between these LMWOGs include Van der Waals, hydrophobic, and hydrogen bonds, which are governed by temperature and shear forces to a large extent. This type of oleogelators includes waxes, fatty acids and alcohol, mono-acylglycerol, ***β***-sitosterol/***γ***-oryzanol, as well as mixtures of LMWOGs such as fatty acids and a fatty alcohol such as lecithin/tocopherol, lecithin/phytosterols, and waxes/monosaccharide [[121], [122], [123]].

##### 3.1.2.2 Waxed-based oleogels

It has been claimed that natural waxes can gel liquid oils at concentrations as low as 1–4% by producing a three-dimensional network that entangles oil inside its pores and by adsorbing oil onto the surface of the network [124,125]. Apparently, wax-based oleogels are produced by heating waxes in liquid oil over their melting point, followed by cooling to 27 °C under shear or quiescent conditions.

The chemical composition of waxes, such as fatty alcohols, fatty acids, and hydrocarbon chains, as well as their origin and relative percentage of these ingredients, have a significant impact on their gelation behavior. Moreover, oil quality significantly influences gelation behavior. Higher saturation levels correspond to improved gelation. Utilizing readily available, cost-effective, and food-grade quality wax-based oleogels, it’s possible to create water-in-oil-type structured emulsions. These emulsions may or may not necessitate emulsifiers. Additionally, their thermo-reversible nature makes them suitable for foods that undergo temperature variations [126]. Candelilla wax (CDW), carnauba wax (CBW), rice bran waxes (RBW), beeswax (BW), and sunflower waxes (FW) are the mostly used food grade waxes used for application in edible oleogel [127]. Ref. [6] created oleogel with canola oil and carnauba wax and used it as a frying medium in the deep-fried chicken sample, which significantly reduced the fat uptake and lipid oxidation.

##### 3.1.2.3 Phytosterols based oleogels

***β***-Sitosterol, a component of most vegetable oils and consequently the most abundant plant sterol, is a sterol that has been studied extensively in the context of oleogelation [128]. ***γ***-Oryzanol, another phytosterol-based gelator, is mostly present in the rice bran as a combination of ferulic acid esters of phytosterols and triterpene alcohols [129]. Individually, neither of these gelators can form a gel, but their synergistic interaction leads to the formation of robust oleogels at concentrations as low as 2%.

The oil-absorbing capacity of formed oleogels is determined by the ratio between ***γ***-oryzanol, ***β***-sitosterol, and vegetable oils, which impacts the crystal structure and, in turn, the oil entrapment rate. A study was conducted on various ratios of ***γ***-oryzanol, ***β***-sitosterol, and canola oil with varied polymorphic forms, melting points, and morphological properties. The authors state that 20%–70% ***γ***-oryzanol, 60%–10% ***β***-sitosterol, and 20%–60% canola oil form a single crystalline phase, which appears to be an effective substitute for trans and saturated fats [130]. The use of surfactants can enhance the microstructural and physical features of oleogels based on phytosterol. According to a recent study, the inclusion of monoglyceride as an emulsifier affects the crystallization behavior of phytosterol-based oleogel [131]. Hydrogen bonding between phytosterols and monoglycerides improves the physical stability and functional properties of the final oleogel [132]. Therefore, it is possible to create versatile oleogels using phytosterols as gelators, considering factors like the appropriate balance between ***γ***-oryzanol and ***β***-sitosterol, the choice of vegetable oils, and the addition of a surfactant to enhance the physical characteristics of the gels.

##### 3.1.2.4 High molecular weight oleogelators (HMWOGs).

HMWOGs are structurants with high molecular mass such as proteins and polysaccharides that are capable of entrapping oil through the formation of a three-dimensional network via hydrogen bonding. The viscoelastic properties of oleogels produced with polymeric oleogelators are significantly affected by the molecular weight, conformation, and concentration of the polymer. Among polysaccharide-based oleogelators are ethylcellulose, methylcellulose, xanthan gum, carrageenan, and chitin [114,133]. Protein-based oleogelators include globular proteins such as egg albumin, β-lactoglobulin, soy protein, and whey protein isolate, and fibrous proteins such as gelatin are commonly used [134]. However, their application to use as a frying medium is limited.

### 3.2 Preparation of oleogel as deep-frying medium

The objective of oleogelation is to provide liquid oil with the characteristics of solid fat without requiring a substantial amount of saturated and trans fats. Consequently, oleogelation aims to develop an alternate approach for structuring, anticipating an edible oil structure that resembles the fat crystal network. Several gelation techniques have been investigated, resulting in oleogels with extremely intriguing features [135], which can be categorized as direct dispersion, biphasic template, and solvent exchange techniques. Among them, direct dispersion techniques have been mostly used for preparing oleogel. In direct dispersion methods, edible oleogels are produced by heating an oil/oleogelators solution above the glass transition (*T*_*g*_) of the polymer and allowing the solution to clarify. Through heating, there are increased polymer/solvent interactions due to the enhancement of the flexibility in polymers. This process facilitates the dissolution of polymers within the solvent. The mixture is cooled below the *T*_*g*_, bringing the polymer to a more rigid state as a result of the formation of hydrogen bonds between the chains of the polymer.

**Fig. 2:**
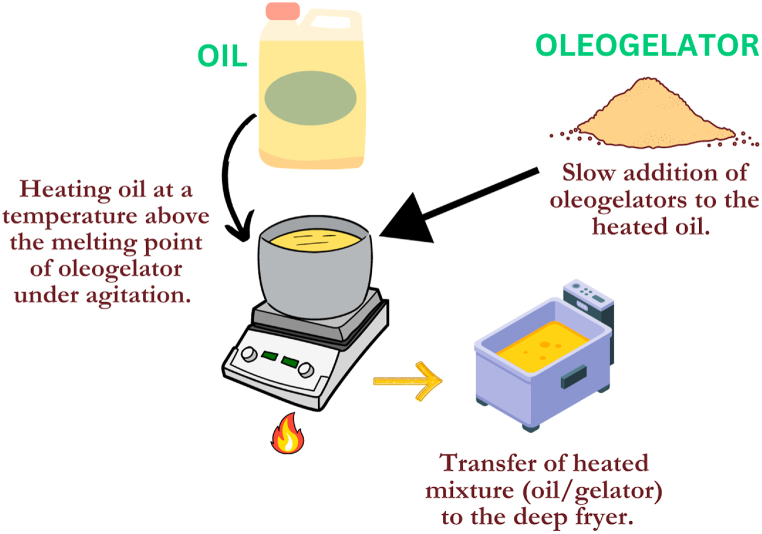
The preparation of an oleogel deep-frying medium.

During the cooling period, inter-and intramolecular forces are established, which leads to the formation of a three-dimensional polymer network that traps the liquid oil. When developing oleogels, an oleogelator material is weighed and added to oil at the desired weight/weight concentration. This mixture is then heated to a temperature above the melting point of the oleogelator material, all while being agitated. Subsequently, the oily dispersion should be cooled to ambient temperature, resulting in the formation of oleogels. Adrah et al. (2022) created oleogel using direct dispersion methods with carnauba wax and canola oil at 5 and 10 g/100 g (w/w) ratio by heating them above 90 °C (melting point of carnauba wax) under continuous agitation (200 rpm) which results in a formation of oleogel after cooling at ambient temperature [6]. The authors used the produced carnauba wax/canola oil oleogel in the deep-fried chicken sample, which greatly reduced fat absorption and lipid oxidation. In the direct dispersion method, the type of structurant used can influence the gelation mechanism, resulting in the formation of two distinct types of networks: crystallite conformations or self-assembled networks [136]. A depiction of the preparation of an oleogel deep-frying medium is presented in Fig. 2. Furthermore, a comparison of typical frying oil and oleogel, based on their different properties, is presented in Table 2.

**Table 2:** Comparison of frying oil and oleogel.

Properties	Traditional frying oils [6,137]	Oleogels for deep frying [6,137]
Source	Plant or animal-based	Plant-based
Composition	Fatty acids, triglycerides, unsaturated/saturated ratio	Structured gelling agents, fatty acids, triglycerides
Solidification temperature	Liquid at room temp	Solid at room temp
Melting point	Varies with oil type	Elevated melting point
Stability	Prone to oxidation	Enhanced oxidative stability
Reusability	Limited (multiple use may degrade oil)	Multiple use with reduced degradation
Smoke point	Varies with oil type	Higher smoke point
Foam formation	Potential for foaming	Reduced foaming
Texture enhancement	N/a	Improved textural attributes
Shelf life	Varies with oil type	Extended shelf life
Crispiness and texture	Consistent results	Enhanced crispiness
Oil absorption	High	Reduced oil absorption
Nutritional content	Varies with oil type	Retains nutritional value
Techno-functional performance	Standard deep-frying characteristics	Improved heat transfer, reduced oil loss

#### 3.2.1 Crystallite conformations

Various types of gelators are capable of generating crystallite conformations. Using a direct dispersion technique, lipid-based gelators, such as waxes, monoacylglycerols (MAGs), fatty acids, and fatty alcohols, produce a crystalline network. This is a highly common method for oleogel synthesis, as it closely resembles the conventional method of oil structure using solid fats. Both processes entail nucleation, crystallization, aggregation, and network creation [138]. However, typical lipid structure is dependent on the creation of triacylglycerols (TAGs) hardstock that, in order to produce efficient oil structuring, must be introduced to the oleogel at a concentration of around 20% [139].

Due to the lower critical concentration required to induce oleogelation, the use of gelators such as waxes, wax esters, and MAGs is a particularly appealing and cost-effective alternative. Typically, just a small purification or concentration step is required to turn these molecules into functional lipid gelators [139]. In addition, the wax content has a substantial impact on the molecular organization of the oleogel structure, making them a viable alternative for a variety of oleogels with vastly diverse thermal and rheological properties. Specifically, the length and degree of saturation of alkyl ester chains influence crystal formation, resulting in alterations at the crystalline network level.

Additional factors, like impurities that influence the shape of wax crystals, and the manipulation of shear and cooling rate, which have demonstrated their effectiveness in altering wax crystal structure, can be employed to tailor the characteristics of the gel for specific uses [140]. Moreover, the configuration and type of crystals formed are also contingent on the chemical makeup of the gelator. Despite similar preparation methods, waxes, monoacylglycerols (MAGs), and other fatty acid derivatives can produce oleogels with unique crystal shapes [141].

#### 3.2.2 Self-assembled network

Essentially four types of gelators can generate self-assembled networks: LMOGs, polymers, colloidal silicon dioxide particles, and lecithin [138,142]. Due to sterols' potential to form very stable oleogels and their health-related benefits, sterol-based oleogels are among the most intriguing and extensively researched LMOGs. Combining oryzanol and phytosterol, for instance, results in a tubular configuration that can generate oleogels with better mechanical qualities [143]. These are referred to as self-assembled fibrillar networks (SAFiNs) because the gelation mechanism involves the production of a collection of fibrils that exhibit unidirectional growth and intertwine to create a fibrillar network. The final length of the fibers is strongly dependent on environmental circumstances such as cooling rate and storage temperature; therefore, it is possible to tailor these parameters based on the desired features of the oleogel [144]. Because these systems create thin fibrils, the structural network is transparent even with high concentrations of the oleogelator. This characteristic allows the oleogel to maintain a similar color to the oil, which can be advantageous for specific applications where the visual appearance of the product is a crucial factor in determining customer acceptability [145]. As per the European Food Safety Authority (EFSA), these substances are not only approved for use in food applications but have also demonstrated the capacity to actively lower blood LDL cholesterol levels and the risk of coronary heart disease. Additional LMOGs, including 12-hydroxystearic acid and ricinoleic acid, serve as oleogelators that can be applied individually or within mixed systems [142,146].

The only known polymer that can function as an oleogelator via the direct technique is ethylcellulose (EC) [144]. After the EC is completely solubilized in the liquid oil at a temperature above the glass transition temperature (140 °C), the polymer softens and there is partial solubilization in the oil. During the subsequent chilling period, the polymer recovers to its rigid state, resulting in the creation of hydrogen bonds and a three-dimensional polymer network. Depending on the viscosity grade, this network imparts greater or diminished viscoelastic qualities to the oleogel. If these conditions are not met, there is a substantial probability of incomplete gelation [135].

Colloidal silicon dioxide (CSD) particles are commonly employed in the food, cosmetic, and pharmaceutical industries [147,148]. They are an approved food additive and have been observed to create gel structures in vegetable oils within oleogel and bigel systems. When incorporated into liquid oil at concentrations of at least 10%, CSD particles assemble into fractal aggregates stabilized by hydrogen bonds and electrostatic interactions [149]. Lecithin is another alternative for the formation of self-assembled networks. When surrounded by a non-polar organic liquid, such as oil, this amphiphilic molecule produces reverse spherical micelles. Lecithin-based oleogels necessitate the addition of a polar solvent (e.g., water); this accelerates the uniaxial growth of the micelles and transforms them into elongated tubular structures that eventually intertwine to form a three-dimensional network. Similar to polymer oleogels, these crosslinked tubules entrap the oil phase and generate a gel [150]. In addition, it has been demonstrated that lecithin can interact with other gelators, such as waxes, phytosterols, and EC, as a co-gelator in direct dispersion methods. In certain cases, the synergistic interactions between the gelators in these multi-component oleogels exhibited superior rheological properties compared to those of single-component oleogels [151,152].

## 4 Characterization of and application of different oleogels in deep-fried food products

### 4.1 Canola oil oleogel

Canola oil is characterized by significant proportions of monounsaturated fatty acids, and polyunsaturated fatty acids including plant sterols (0.53–0.97%), tocopherols (700–1200 ppm), 21% linoleic acid, 11% alpha-linolenic acid, 61% oleic acid, and a low content (7%) of saturated fatty acids (Lin et al., 2013). The quality of canola oil declined significantly during cooking at high temperatures [153], but remained stable in oleogel systems [154].

A study by Ref. [155] concluded that onion and potato fried in sesame oil was better than canola oil at 180 °C for 8 and 4 min, which agrees with the observation that canola oil degrades rapidly under high temperatures [153]. When entrapped in a composite three-dimensional system, canola oil-carnauba wax oleogel improved the texture, color, and reduced fat uptake in fried chicken nuggets. These effects were ascribed to the compact nature and the oil-binding capacity, and the synergistic effects of the gel-forming material, which reduced moisture loss through evaporation, minimized crust and pore formation, and consequent fat migration into the fried product. According to Ref. [156], the distinctive texture of fried products directly correlates with its frying medium.

In their subsequent investigation, [6], revealed that oleogel-fried chicken had a lower fat uptake of 8.53% compared to canola oil-fried samples (Table 3). More interestingly, the oxidation and thiobarbituric acid reactive substances were reduced and the moisture content was much higher in the samples fried in oleogel than in the canola oil-fried samples. Given these satisfactory results, further exploitation of this novel technique will provide a plausible alternative and possible solution to solid fat usage in the food industry.

**Table 3:** Application of oleogel as a frying medium in different deep-fried food products.

Product	Gelator/Oil Matrix	Gelator Conc.	Outcomes	Refs.
Chicken breast	Carnauba wax/Canola	5%/10%, (w/v)	Samples fried in 5% and 10% showed lower fat absorption – 8.53% & 9.15%, respectively. Samples fried in 5% oleogel had the lowest TBARS. Observable microstructural differences between only canola oil and oleogel fried samples.	[6]
Chicken breast	Carnauba wax/Canola	5%/10%, w/v	Oleogel-fried samples had significantly lower fat content. In combination with Thyme Essential Oil (TEO), oleogel-fried samples had improved oxidative stability.	[154]
Instant Noodles	Carnauba wax/Palm oil	5%/10%, w/w	Reduced fat uptake and improved cooking properties. Smoother microstructural surfaces and more retention of unsaturated fatty acids than only palm oil fried samples. Better storage quality in terms of TBARS, PV, p-AnV, and TOTOX values at room temperature for 12 days.	[157]
Indian Traditional product (Mathri)	Carnauba wax/Soybean oil	5, 10 &15%	In comparison to Mathri cooked in soybean oil, Fried mathri prepared using oleogel exhibited enhanced moisture retention, color, and texture, while displaying reduced oil absorption. Specifically, the breaking strength of mathri in regular oil was 3382.1 g. In comparison, mathri fried in oleogel recorded breaking strengths of 1974.8 g (5% oleogel), 1092.7 g (10% oleogel), and 3168.1 g (15% oleogel). Moreover, mathri fried in oleogel exhibited a notable reduction in oil absorption, with 27.7%, 22%, and 19.3% less oil absorbed compared to those cooked in regular oil.	[158]
Instant fried noodles	Carnauba wax/Soybean oil	5%/10%, w/v	The samples fried in oleogels took up approximately 16% less oil than the palm and soybean oil-fried noodles, as confirmed by scanning electron microscopic images. The texture of the noodles remained unaffected. In comparison to noodles fried in palm oil, the oleogel-fried noodles contained significantly lower levels of saturated fatty acids, specifically 19%. Moreover, the samples displayed reduced peroxide levels during storage.	[111]
Potato strip	Beeswax/Sunflower oil	3%/8%, w/v	Compared to the control sample (only sunflower oil cooked), potato strips cooked in oleogels absorb considerably less oil. Additionally, oleogel-fried potatoes are brighter and yellower than the control sample. The samples cooked in oleogels are tougher, springier, and gummier than the control sample, according to the fried potato samples' textural character. Oleogel-fried potatoes have higher sensory scores, according to a sensory study.	[66]
Onion ring	Polyglycerol stearate/Sunflower oil	3%/8%, w/v	The onion ring samples that were cooked in oleogels absorbed around 33–37% less oil compared to the control group. The L* value, aroma, crispness/texture, and overall acceptability scores for the onion ring samples that were fried in the oleogels did not suffer as a result of their use.	[159]

### 4.2 Corn oil oleogel

Corn oil, with its distinctive clear pale yellow color and a high proportion of polyunsaturated fatty acids, are a few attributes contributing to its utilization in the deep frying industries [160]. Corn oil has been combined with different biopolymers in oleogel formulations and characterized for their suitability as fat replacers in food products [161]. Different gelation techniques were employed to gel corn oil with monoglyceric stearate, β-sitosterol/lecithin, and ethylcellulose as partial or whole replacements for regular butter in chocolate. A high degree of unsaturation and shear thinning of corn-based oleogel was found in the chocolate products. It was inferred that the thermal characteristics of the oleogel based product similar to dark chocolate could warrant their preference for butter in chocolate production [162]. High-intensity oscillatory stress analysis revealed that the network structure and nonlinearity of the corn oil-oleogel were less distorted [163]. The study further showed that corn oil blends entrapped in zein-based oleogel displayed malleability and thermal reversibility and could be a useful tool for active ingredient delivery. In other studies, corn oil–based oleogel structured with different concentrations of β-sitosterol and γ-oryzanol/lecithin/stearic acid produced a strong gel with a dense-crystallization network. The gel system displayed desirable morphological, texture, rheological, and oil binding capacity, which was linked to the robust internal three-dimensional framework formed by the gel [164]. In general, corn oil oleogel has been successful in the bakery industry, but with very limited application in the deep-fat frying sector.

### 4.3 Sunflower oil oleogel

Sunflower seed oil, among the various vegetable oils, is the most commonly used in the food industry. The oil is known for its superior sensory properties and remarkable antioxidant properties [165]. The oil content of commercially available sunflower varieties ranges from 39% to 49% [165].

Oleogels and biphasic structuring networks from sunflower oil can be obtained with different oleogelators with desirable characteristics. For instance, Ref. [166], developed and characterized sunflower oil oleogel loaded with regenerated chitin as the stabilizing particles. The viscoelastic properties of the composite gel were significantly improved. Incorporation of chitin at different concentrations (0.8/100–1.4 g/100 g) exhibited strong resistance against coalescence and Ostwald ripening during the storage time. The author also revealed that the oleogel presented great thermal stability against oil leakage when heated to 80 °C for 2 days.

Their high amounts of poly and monounsaturated fatty acids (liquid nature) could facilitate their efficient encapsulation in a polymer matrix. The differences in the level of unsaturation in sunflower oil and the other tested vegetable oils had a significant impact on the strength, solid fat content, and morphology of the sunflower-wax-based oleogel systems. These oleogels exhibited product stability concerning storage, particle size, and an oil binding capacity exceeding 90% [166]. The study further revealed that the oleogel presented greater intrinsic elastic characteristics and less spreadability, which was ascribed to the bulk properties of the gel.

Although there is limited literature on sunflower oil oleogel in deep-fat frying, a few studies have demonstrated their characteristics affect fried products. Ref. [159], prepared a gel sunflower oil with different proportions of polyglycerol stearate oleogels as an alternative deep-fat frying medium for onion rings. A significant enhancement in the organoleptic and consumable attributes was recorded in the oleogel-fried samples. The authors further revealed that the onion ring samples fried in oleogel showed 33–37% less oil absorption, compared to the control group (Table 3). It has been suggested that the oil absorption inhibition effect and the sensory retention capacity of sunflower oil oleogel could play a crucial role in reducing total calories and weight gain in consumers.

### 4.4 Peanut oil oleogel

The vast cohort studies on peanut oil and its wide application in food systems are partly linked to its appealing sensory characteristics and bioactivity [167]. The characterization of the oxidation stability of oleogel from peanut oil and soybean oil incorporated with blueberry extracts and resveratrol showed that the gel system could inhibit primary and secondary oxidative products for 30 days [168]. The morphology, surface properties, and microrheological analysis of peanut oil with yellow beeswax oleogel demonstrated an increase in firmness, elasticity, firmness, and resistance to the centrifugal force in the gel system [169]. It was speculated that the long-chain fatty acids present in peanut oil positively influenced the wax crystal network and thus enhanced the gel stability. The increased elasticity and viscosity were attributed to the compact and constrained particle mobility by the three-dimensional network generated by the oleogel.

A study by Ref. [170] investigated the comparative effects and physical characteristics of peanut oil and soybean oil oleogel as a delivery system for resveratrol. Considerable thermal stability, gel strength, and sensory attributes were noticed in both systems, but the physical characteristics of peanut oil oleogel remained stable over prolonged storage. The authors concluded that the oleogels have the potential to serve as a delivery system for active ingredients. As different vegetable oils are structured with different gelators as a substitute for saturated fats and to reduce oxidation drawbacks with liquid oils, whether peanut oleogels could fulfill these requirements needs further elucidation.

### 4.5 Soybean oil oleogel

Soybean contains about 18-22% oil, with 85% unsaturated fatty acids [171]. Similar to sunflower oil, the presence of a significant amount of unsaturated fatty acids renders soybean oil susceptible to oxidation during frying [172], which may result in greasy, less crispy, and reduced shelf stability of the product [111] (Table 3). Preceding studies have demonstrated that immobilization of soybean oil in a biphasic system leads to physical entanglements without affecting the chemical properties of the oil [173]. It was found that increasing the concentration of oleogelators increased the hardness, smoke point, and oil binding capacity of soybean-carnauba wax oleogel from 0.05 to 23.6 N, 232 °C to 283 °C and 68.76–99.73%, respectively [173]. Indoria, Solanki, and Meena, (2017) reposed that the increase in smoking points is directly related to the reduction in free fatty acids in the oil. In addition, the number of crystals present in the oil can modify gel shape and hardness [174]. A study by Ref. [175] examined the rheological, texture, microstructure, and oil-binding properties of soybean oil-based oleogel. The authors observed a sheet-like and needle-like structure and droplet of oil entrapped in the oleogelator, demonstrating efficient oil encapsulation. The increase in the gel hardness and oil-retention ability was ascribed to the synergistic effect of the composite system, which resulted in a bulk gel network to reduce micropores in the oleogel.

A comparative study was carried out on palm oil and soybean oil–based oleogel as frying media for instant fried noodles. As expected, the oleogel-fried samples exhibited reduced oil uptake and low saturated fatty acid composition compared to palm oil. During the frying process, water present in the foods transforms into steam and escapes, leading to the development of a porous structure. As a result, vented holes were clearly visible on the surface of the noodles. However, it was observed that noodles fried in palm and soybean oils exhibited large blisters, causing a rough and coarse texture. In contrast, the noodles fried using oleogels displayed a consistently smooth surface. These distinct surface features may be linked to variations in oil uptake by the noodles, as higher surface roughness tends to increase the amount of oil adhering to fried foods. It was further noticed that the samples fried in the oloegel increased firmness and peroxidase activities. The dramatic rise in the peroxidase values of samples fried in soybean oil-based oleogel was attributed to the unstable nature and sensitivity of soybean oil to oxidation. Nonetheless, it was suggested that increasing the concentration of encapsulating agents may mitigate these drawbacks [111].

### 4.6 Palm oil oleogel

Palm oil, with a low melting point (22–24 °C), is highly stable during frying owing to its antioxidant and fatty acid content [176]. Palm oil is commonly mixed and esterified with other oils or biopolymers to create margarine and composite frying media [177]. Compared with palm fat, candelilla wax, rice bran wax and yellow bees wax-based biphasic systems exhibited a product gel structure, appearance, and strong oil binding capacity [178].

According to Ref. [179], the crystal morphology, microrheological properties, and physical stability of palm oil can be improved when fortified with excellent gelling biopolymers. Carnauba wax at varying proportions (5 g/100 g and 10 g/100 g, w/w) was employed to encapsulate palm oil for the deep frying of salted duck egg white-fortified instant noodles (Table 3). Compared with oleogel-fried noodles, the sample fried in palm oil only exhibited uneven and rough microstructure which was ascribed to the induced complex interaction between the protein content and gelatinized starch. The rough surface morphology further contributed to higher oil absorption in the samples. The tensile properties, physiological and sensory attributes of the noodles fried in the oloegel system were considerably better than palm oil samples. The results further proved that the oleogel-fried samples presented higher oxidation stability, and lower TBARS during ambient storage for 12 days. This comparative study showed that blending palm oil with different oloegelators could improve their characteristic effects, and stability and enhance their application in the food industry [157].

### 4.7 Rice bran oil oleogel

Rice bran oil is known for its active components such as tocopherol, squalene and superior oxidative stability, compared with other vegetable oils [180]. The oil is widely used as a frying medium, given its rich source of monounsaturated fatty acids, sterols, and cardiovascular disease preventive ability [180]. For better functionality and stability in food applications, rice bran oils are modified to mimic solid fats.

The physical and viscoelastic characterization of oleogels containing 0.5–25 wt% rice bran wax in rice bran oil was explored. The wax crystal resisted phase transition under thermal conditions, increased the gel viscosity with cooling, and displayed maximum oil retention [181]. Compared with flaxseed oil-based systems, oleogels from rice bran oil exhibited higher gel firmness, great oil binding capacity, and reduced oxidation activities [182]. The alteration in microstructural and physicochemical properties of oleogels from candelilla wax and rice bran oil added with differently sourced lecithin was studied [183]. The microstructural analysis revealed a variety of globular and granular fat formations with varying sizes, densities, packing, and brightness, which was attributed to decreased fat crystal aggregate by the added emulsifying agents. The authors noticed that the gel showed bright micrographs with better packing and spreadability. The extraction of crude rice bran wax as an olegelator for rice bran oil is not only a novel technology in the food industry, but it reduces waste and enhances the underused product [184]. Oleogels from rice bran have limited application in deep frying. Thus, future studies should ascertain their effect as a potential substitute for trans fatty acids.

## 5 Health impact, oxidative stability, and sensory acceptability

Due to its desirable characteristics, such as simple production, excellent fatty acid content, and safe application in food products to suit customers' needs for healthy products, oleogel has attracted growing interest [185]. By incapacitating liquid edible oils in a 3D network created by an oleogelator, oleogels offer the opportunity to swap out traditional saturated fatty acid (SFA)-based lipids for a healthier substitute.

### 5.1 Health impact

In a study examining the health benefits of oleogel made from rice bran wax in rice bran oil, rats that were given oleogel experienced significant reductions in the formation of fat tissue, as well as decreased levels of total cholesterol in the liver and both blood and liver triacylglycerols, when compared to rats that were fed beef tallow and commercial margarine. Moreover, the oleogel resulted in higher levels of total cholesterol, bile acid, and triacylglycerols being excreted in feces. Recent research indicates that the overall positive health effects of oleogel stem from a combination of factors, including substituting unhealthy fats with healthier oils, the actions of the oleogelator itself, and the gel’s structure, which controls the release of lipids into the bloodstream [186].

According to a specific study, the utilization of an oleogelator on its own can potentially offer health benefits. For example, in research conducted with sunflower wax, identified as a potential oleogelator, it was found that rats that were fed diets containing 0.5% and 1.0% of this pure wax experienced a 21% and 22% reduction in their total cholesterol levels compared to rats on standard diets [187]. Because sunflower seeds and their oil contain approximately 2–2.5% and 0.02–0.35% wax, respectively, and are consumed by individuals, this research was conducted to evaluate the potential nutritional effects and toxicity of the wax [188]. In another study, beeswax and carnauba wax were examined for their effects on adipogenesis in 3T3-L1 cells. It was shown that these waxes dramatically reduced the lipogenesis pathway by controlling the expression of adipogenic genes, which had an obesogenic impact [189]. In other research, the impact of oleogel on health was compared to that of comparable oil. In comparison to an oil-water combination, an oil-water-monoglyceride oleogel dramatically reduced free fatty acids, insulin levels, and blood triglyceride in people after acute ingestion [190].

Another study showed that consuming coconut oil in the form of oleogels instead of normal oil significantly reduced the after meal blood triglyceride level in adults [191]. Similar to this, in simulated intestinal lipolysis, canola oil oleogels produced with ethyl cellulose and the combination of β-sitosterol and γ-oryzanol demonstrated slower lipolysis expressed as the total free fatty acids released than canola oil [12]. In a separate study, rats were divided into two groups: one group received an oil blend, while the other group was provided with oleogels created using a (15 wt%) mixture of either palm stearin and cetyl laurate or palm stearin and cetyl caprylate in rice bran and flaxseed oils. The rats that consumed the oleogels exhibited reduced cholesterol levels as determined by an analysis of their lipid profile, with a more pronounced cholesterol-lowering effect observed when using palm stearin and cetyl caprylate in comparison to palm stearin and cetyl laurate [192]. Because of the action of the oleogelator itself and the gradual release of oil into the circulation, oleogels may offer additional health advantages over and beyond the fundamental advantages of replacing or changing bad fats with product vegetable oil. Oils including phytosterols, omega-3 fatty acids, flavonoids, and other competent components are frequently added to products to further increase the health benefits of oleogel [193].

### 5.2 Oxidative stability

Due to the oil being immobilized in the gel structure, as previously indicated, oleogels may oxidize more slowly than bulk oil. It should be emphasized that the major objective of using oleogels is to replace unhealthy oils high in unsaturated fatty acids, which are considerably more susceptible to oxidation than saturated fatty acids, with fats rich in saturated fatty acids. Therefore, the oxidative stability of oleogels and food products containing oleogels must be investigated. If required, specific precautions like more antioxidants must be taken to stop oleogels from oxidizing and the harmful health repercussions that follow [194]. Numerous investigations demonstrated that diets containing oleogel have enough oxidative stability without the addition of extra oxidants. For instance, in a recent study, samples of margarine were manufactured with a high-oleic sunflower oleogel made with skim milk, salt, and additional additives often found in margarine, such as butter taste, an antioxidant called potassium sorbate, and beta-carotene. Even though the oleogel-based margarine included more unsaturated fatty acids, its oxidative stability was comparable to that of commercial margarine and was acceptable for 6 months of storage at 5 °C [195].

However, some studies claimed that oleogelators have prooxidant action. For instance, it was noted that the carnauba wax-formed cod liver oil oleogel oxidized more quickly than the control oil, showing that carnauba wax enhanced oil oxidation [196]. The same study, however, demonstrated that beeswax did not encourage oil oxidation. Different research, however, found that both oleogels had stronger oxidative stability than grapeseed oil and that carnauba wax provided grapeseed oil oleogels with better oxidative stability than beeswax [197]. The varying wax purity resulting from various suppliers and/or purifying techniques may have contributed to the inconsistent results. Another study [194] claimed that small wax components might have prooxidant effects. Due to the fact that natural sources of oleogelators contain a variety of small components that may have prooxidant activity, one should exercise caution while choosing them. In a study, using the same oleogel, conflicting outcomes were also discovered when several analytical techniques were employed.

The oxidative stability of fish oil oleogel made from a combination of sorghum bran wax, sorghum distillers’ dried grains and soluble (DDGS) wax, and sorghum kernel wax was investigated [78]. This oleogel exhibited a higher Peroxide Value (PV) but lower p-Anisidine Value (p-AV) when compared to the control fish oil. This highlights the variability in findings that different analytical methods can yield. Assessing the oxidative stability of an oleogel requires employing multiple analytical approaches. Initially, primary oxidation products such as hydroperoxides and conjugated dienes emerge during the oil’s oxidation process. Subsequently, these elementary oxidation products break down or react with other compounds, giving rise to an array of secondary oxidation products, including aldehydes, ketones, alcohols, epoxides, polymers, and other substances [198]. Discrepancies in results frequently arise from using various analytical techniques for oil oxidation because there are numerous oxidation products generated during this process, and each analytical method detects only one specific type of oxidation product [198]. Therefore, the most prudent approach for now is to monitor at least two or three oxidation products simultaneously, unless a technology is developed that enables the concurrent detection of both primary and secondary oxidation products.

**Fig. 3:**
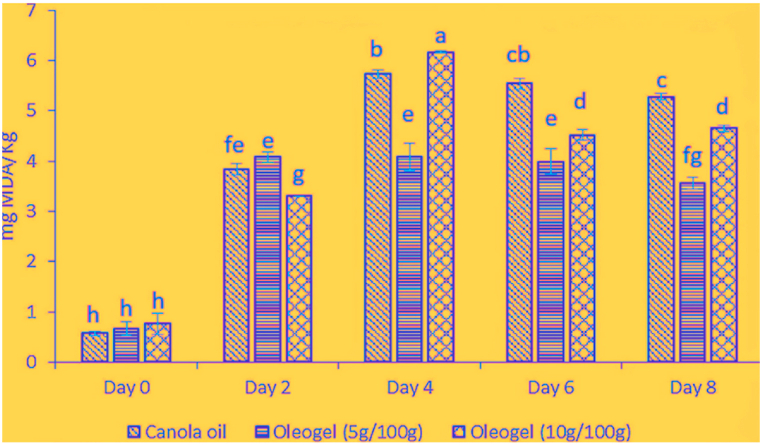
Changes in TBARS values of chicken breast samples deep-fried in canola oil and oleogels (5% & 10%) [6].

Studies relating to the oxidative stability of oleogel and prepared products that are subjected to deep-frying are still scarce. A recent study investigated the lipid oxidation of oleogel-deep fried chicken products using TBARS during 8 days of storage [6]. Lower MDA levels were detected in samples that had been fried in oleogels, particularly toward the end of the storage period (Fig. 3). All samples' measurements of thiobarbituric acid reactive compounds on successive days rose until day 4 when a slight drop was noticed. The cause can be connected to the early formation of MDA’s potential breakdown [199]. With the exception of the 10% oleogel-fried samples on day 4, the degree of oxidation of chicken breast samples was much lower in oleogels than in canola oil. According to another research, the effectiveness of oleogels in postponing lipid oxidation may be attributable to the oil’s constrained mobility as a result of their firm structure. The 3-dimensional gel structure traps the oil, delaying air oxidation [11,200]. As previously indicated, research on oleogel oxidative stability during deep-frying is still limited. Understanding the variations in oleogel quality during frying and oxidative stability requires more investigation.

### 5.3 Sensory acceptability

Although many oleogelators and associated food products have been researched, only those with acceptable sensory qualities may be marketed. Recent research assessed the sensory qualities of products containing oleogel. The sensory evaluation of 120 panelists' responses to samples of margarine containing a high-oleic sunflower oleogel, made with a combination of candelilla wax and monoacylglycerols, revealed that consumers' intentions to purchase the oleogel margarine were comparable to those of the commercial variety, despite the latter’s lower ratings for appearance, color, aroma attributes, overall taste, texture, and impression [195]. The use of oleogel as a frying medium for deep-fried foods and subsequent assessment of sensory acceptability are still in their infancy. Only a few research have looked at the sensory characteristics of products that have been oleogel-fried. For instance, a recent study examined the effects of using oleogel as a frying medium to create low-fat versions of Indian traditional food (Mathri). According to the study, samples that were 10% oleogel fried received the best marks for aesthetics. Similar findings were obtained regarding color, where samples that had been fried at a 10% concentration had the best color (high L* and low b* values). The samples that were fried in 5% oleogel received the lowest rating. The highest scores for crispiness were seen in samples that had been fried in oleogel which was 15%. Contrary to what the instrumental measurements showed, customers liked the 15% oleogel samples' crispiness above those that had been cooked in oil. The least crispiness was found in samples that were fried with 10% oleogel, which is comparable to the value for instrumental texture. The samples that were cooked in oil and those that included 10% oleogel were thought to be superior to the other two in terms of overall acceptability and flavor. The sensory panelists claimed that samples prepared with a greater oleogel content (15%) had a waxy feel and flavor, which is why they received the lowest scores. Because of this, the amount of oleogelator utilized in the manufacture of the oleogel is important in terms of sensory acceptability [158].

Another study made sunflower oil-beeswax oleogels at concentrations of 3% (BWO-3) and 8% (BWO-8) organogelator to compare oleogels to commercial sunflower oil (SO) as a frying medium for potato strips [66]. They investigated sensory acceptability as well. The results showed that the BWO-8 sample had the highest color score (8.95). Fried foods have a very distinct fragrance, and once more, the sample that had been fried in BWO-8 had the best smell. According to sensory crispness results, oleogel-fried potatoes are crispier, and as oleogels get more solid (BWO-8), so does the degree of fried potato crispness. Potatoes cooked in BWO-8 medium had a better flavor than the control sample. This distinction must be the result of interactions between various frying oil ingredients and the beeswax used to make oleogel as well as the frying process itself. Compared to the control sample, potatoes fried in oleogels scored higher overall for acceptability. Most notably, because oleogel (a sample containing 8% beeswax) is more solid, the potato that is fried in it receives higher sensory ratings [66]**.**

Numerous studies have underscored the significant potential of oleogels as healthier fat alternatives in food products, leading to a substantial surge in interest in oleogel technology for the food industry. Nonetheless, this innovative approach has yet to gain widespread adoption. To bridge this gap, further research is imperative, particularly to deepen our understanding of the factors influencing gel properties, the interplay between oleogelators and food ingredients, and the integration of oleogels into a wider array of food products. Recent investigations have hinted at the potential of blending oleogelators to enhance gel properties when compared to single oleogelators, thereby suggesting the necessity of exploring binary and ternary systems for superior oleogelator combinations. While a few studies have begun to compare different oleogelators, more comprehensive research is required to aid in the selection of the most suitable oleogelator for specific applications. Moreover, the composition of minor components within oleogelators and oils significantly impacts gel properties, further complicating the quest for the optimal oleogelator for particular food products. It’s important to note that some oleogelators can negatively impact sensory attributes, making it critical to consider their effect on the final product’s sensory qualities when choosing an oleogelator. Research findings strongly suggest that partially substituting traditional animal fats like beef tallow and lard with oleogels in meat products holds great promise.

## 6 Conclusion and future perspectives

Several investigations on fat reformulation have been conducted in response to recent policy changes mandating the removal of trans fats from food items and imposing restrictions on saturated fat consumption. These studies are also driven by consumers' increasing concerns regarding the adverse effects of fats and the environmental consequences of excessive cooking oil usage. Innovations in reformulating fat-containing food products are primarily based on incorporating oleogels into the food matrix or developing blends that combine the original fat source with new oleogels.

However, despite promising results and formulations, a variety of oleogelation techniques and organogelators, similarities in physical properties between oleogels and conventional fat-containing food products, nutritional value offered by the use of vegetable oils, and positive outcomes from sensory analysis and hedonic tests on food products with oleogels, commercially available food items with oleogels do not currently exist. To improve the acceptance and consumption of food products incorporating oleogels, it is crucial to explore how various processing parameters unique to specific food items affect oleogel properties. Furthermore, advocating the advantages of oleogel usage to food producers and highlighting the nutritional and environmental benefits are essential. Additionally, future study is required to improve the technical functionality and sensory acceptance of oleogel-based solutions. Only a small number of the oleogelators now in use are GRAS-qualified, hence it is essential to investigate various GRAS oleogelators for their prospective usage in products. To increase the shelf life of products made from oleogel, more natural antioxidants should be added, and storage stability studies of the products should be widely conducted. There have been a few studies on their creation, manufacture, and characterization, but there are currently few that look at how oleogel degrades after digestion. With this method, oleogel-based systems may be used to release bioactive substances into the human gut. Although oleogel-based frying media has been successfully used in deep-frying, there are currently no commercial applications. In order to assess the viability of using oleogel in deep-frying, additional study is required to fully comprehend the mechanism, stability of oleogel, sensory impact, and a holistic evaluation of physicochemical testing. This will enable researchers to act pragmatically and devise strategies for putting oleogel into context and commercializing them.

## Funding

This project received financial support from the National Institute of Food and Agriculture of the United States Department of Agriculture.

## Data availability statement

The data that support the findings of this study are available within the article.

## CRediT authorship contribution statement

**Niaz Mahmud:** Methodology, Software, Writing – original draft. **Joinul Islam:** Methodology, Software, Visualization, Writing – original draft, Writing – review & editing. **William Oyom:** Software, Visualization, Writing – original draft, Writing – review & editing. **Kelvin Adrah:** Software, Visualization, Writing – original draft, Writing – review & editing. **Samuel Chetachukwu Adegoke:** Software, Visualization, Writing – original draft, Writing – review & editing. **Reza Tahergorabi:** Conceptualization, Data curation, Investigation, Methodology, Project administration, Supervision, Writing – original draft, Writing – review & editing.

## Declaration of competing interest

The authors declare that they have no known competing financial interests or personal relationships that could have appeared to influence the work reported in this paper.
